# Diagnostic challenges in ornithine transcarbamylase deficiency lacking genetic confirmation: liver biopsy *versus* human induced pluripotent stem cell technology

**DOI:** 10.1016/j.ymgmr.2025.101239

**Published:** 2025-06-26

**Authors:** Alexander Laemmle, Niklas Naef

**Affiliations:** a Division of Pediatric Endocrinology, Diabetology and Metabolism, Department of Pediatrics, Inselspital, Bern University Hospital, University of Bern, Bern, Switzerland; b University Institute of Clinical Chemistry, Inselspital, Bern University Hospital, University of Bern, Bern, Switzerland; c Department of Biomedical Research, University of Bern, Bern, Switzerland

**Keywords:** Urea cycle disorder, Ornithine transcarbamylase deficiency, Induced pluripotent stem cell technology, X-chromosomal inactivation

To the Editor,

Further to our recent case report describing the use of human induced pluripotent stem cell (hiPSC) technology to substantiate the diagnosis of ornithine transcarbamylase (OTC) deficiency (OTCD) in a female patient (designated OTCD_4) lacking genetic confirmation [1], we herewith report subsequent findings that reinforce the diagnostic complexities of OTCD, particularly in heterozygous females.

We subsequently analyzed OTC enzyme activity alongside Western blot analysis on tissue homogenates across multiple samples from different liver segments of the OTCD_4 patient's explanted liver (segments 5, 6, and 7).

Our analysis of the explanted liver revealed marked heterogeneity in both OTC activity and protein levels (Fig. 1). We observed substantial variability not only *between* anatomical segments but also *within* samples derived from the same segment. For example, measurements within segment 6 ranged from severely deficient (approx. 35 mU/mg protein; reference value >160) to normal activity (approx. 210 mU/mg protein), with corresponding variations in Western blot analysis. This pattern underscores the mosaic nature of hepatic OTC expression resulting from random X-chromosomal inactivation (XCI) in this female patient. The existence of such cellular mosaicism comprising patches of OTC-positive and -negative hepatocytes was first visualized histochemically by Ricciuti et al. in their foundational study confirming the X-linkage of OTCD [2].

**Fig. 1:**
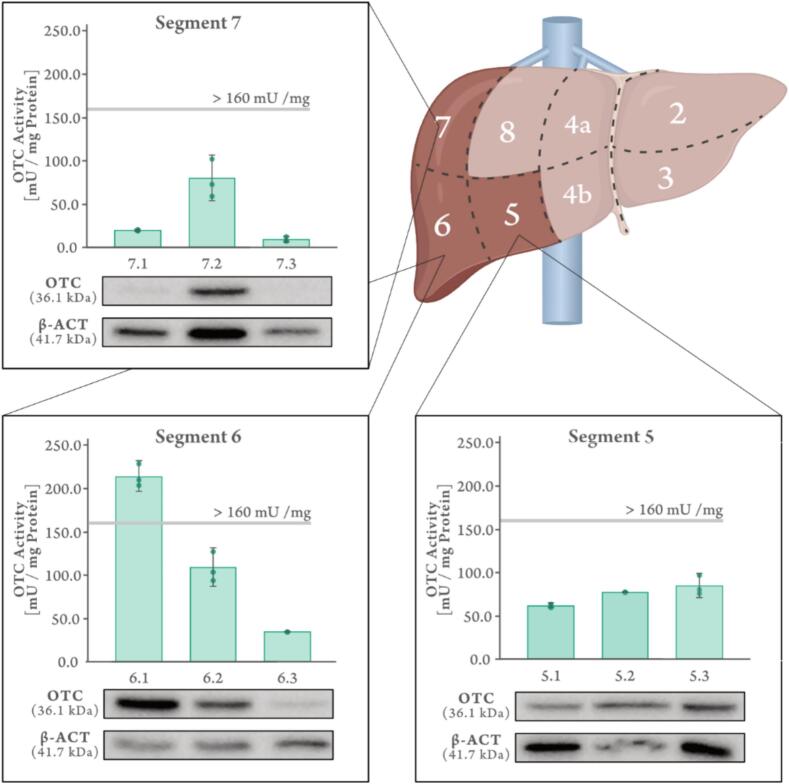
Heterogeneity of OTC activity and protein expression in the explanted liver of patient OTCD_4. Bar graphs represent the mean of three independent measurements. Error bars represent the standard deviation (SD).

These *in vivo* findings from the patient's explanted liver provide direct evidence for a critical limitation of relying on liver biopsy for OTCD diagnosis in heterozygous females. The significant spatial variability means that a single needle biopsy could yield highly variable and potentially misleading results depending on the precise sampling location, potentially leading to diagnostic errors. This inherent limitation of liver biopsy, coupled with the known diagnostic gap in molecular genetic testing (affecting up to 20 % of cases [3]), highlights the need for alternative diagnostic strategies.

The observed hepatic mosaicism strongly supports the conclusions of our initial report [1] regarding the value of hiPSC technology. Deriving several hiPSC clones from patient fibroblasts, as performed by Ramosaj et al. [1], allows for the isolation and analysis of distinct cell populations reflecting the underlying XCI mosaicism. Subsequent differentiation into hiPSC-derived hepatocytes enables the *in vitro* assessment of the spectrum of cellular phenotypes resulting from this mosaicism. This clonal analysis approach, as we demonstrated [1], offers a less invasive method (requiring only a skin biopsy) to assess the functional impact of XCI mosaicism, providing a potentially more reliable diagnostic picture than a single liver biopsy.

In conclusion, the analysis of the explanted liver from patient OTCD_4 provides direct *in vivo* confirmation of the significant hepatic mosaicism in female OTCD carriers and the consequent unreliability of liver biopsy for accurate diagnosis. These data further strengthen the rationale presented in our previous work [1] for utilizing hiPSC-based models as a valuable diagnostic tool in challenging cases of suspected OTCD lacking genetic confirmation.

## Informed consent and ethical approval

Written informed consent was obtained from the patient. This study was approved by the local ethics committee in Bern, Switzerland (project ID: 2020-02979).

## CRediT authorship contribution statement

**Alexander Laemmle:** Writing – review & editing, Writing – original draft, Methodology, Investigation, Formal analysis, Data curation, Conceptualization. **Niklas Naef:** Writing – original draft, Methodology, Formal analysis, Data curation, Conceptualization.

## Declaration of competing interest

The authors declare no competing interests.

## Data Availability

Data will be made available on request.
